# Reproducible and label-free biosensor for the selective extraction and rapid detection of proteins in biological fluids

**DOI:** 10.1186/s12951-015-0102-8

**Published:** 2015-06-24

**Authors:** Arumugam Sivanesan, Emad L Izake, Roland Agoston, Godwin A Ayoko, Martin Sillence

**Affiliations:** Nanotechnology and Molecular Sciences Discipline, Faculty of Science and Engineering, Queensland University of Technology, 2 George St., Brisbane, QLD 4001 Australia; Discipline of Biosciences, Faculty of Science and Engineering, Queensland University of Technology, 2 George St., Brisbane, QLD 4001 Australia

**Keywords:** Aptamer-functionalized biosensor (aptasensor), Nanosensor, Homogenous SERS, Wide area illumination, Erythropoietin (EPO), Horse plasma

## Abstract

Erythropoietin (EPO), a glycoprotein hormone of ∼34 kDa, is an important hematopoietic growth factor, mainly produced in the kidney and controls the number of red blood cells circulating in the blood stream. Sensitive and rapid recombinant human EPO (rHuEPO) detection tools that improve on the current laborious EPO detection techniques are in high demand for both clinical and sports industry. A sensitive aptamer-functionalized biosensor (aptasensor) has been developed by controlled growth of gold nanostructures (AuNS) over a gold substrate (pAu/AuNS). The aptasensor selectively binds to rHuEPO and, therefore, was used to extract and detect the drug from horse plasma by surface enhanced Raman spectroscopy (SERS). Due to the nanogap separation between the nanostructures, the high population and distribution of hot spots on the pAu/AuNS substrate surface, strong signal enhancement was acquired. By using wide area illumination (WAI) setting for the Raman detection, a low RSD of 4.92% over 150 SERS measurements was achieved. The significant reproducibility of the new biosensor addresses the serious problem of SERS signal inconsistency that hampers the use of the technique in the field. The WAI setting is compatible with handheld Raman devices. Therefore, the new aptasensor can be used for the selective extraction of rHuEPO from biological fluids and subsequently screened with handheld Raman spectrometer for SERS based in-field protein detection.

**Electronic supplementary material:**

The online version of this article (doi:10.1186/s12951-015-0102-8) contains supplementary material, which is available to authorized users.

## Background

Erythropoietin (EPO), a glycoprotein hormone of ∼ 34 kDa, is an important hematopoietic growth factor, mainly produced in the kidney and controls the number of red blood cells circulating in the blood stream [1]. After the establishment of human EPO gene sequence [2], recombinant human EPO (rHuEPO), a structural and bio-active analogue of human EPO, has been produced as a pharmaceutical to treat patients suffering from anaemia symptoms associated with various disorders such as cancer [3]. rHuEPO has also been used by athletes as a doping agent in endurance sports to enhance their performance [4]. This prompted World Anti-Doping Agency (WADA) to ban the use of the drug in sports activities [1]. In addition to its chief function in promoting erythropoiesis, it was recently indicated that EPO levels in cancer patients, especially when receiving chemotherapy, may significantly affect the growth and progression of malignant tumours [5, 6]. Thus, sensitive and rapid rHuEPO detection tools are in high demand for both clinical and sports industry [7].

In recent years, SERS has emerged as an ultra-sensitive analytical tool [8–10]. The two important features for real world applications of SERS are the homogeneity of the SERS substrate and selectivity towards target [11]. Vast numbers of SERS active surfaces comprising various roughened metallic surfaces and noble metal nanostructures have been produced. However, the majority of surfaces failed to produce a homogeneous SERS signal. Conversely, very few substrates which are promising to deliver homogeneous and reproducible signal are expensive [12]. Therefore, there is a high demand for cheap, homogeneous and reproducible SERS substrates. In principle, the basic requirement for a homogeneous and sensitive SERS substrate is defect-free arrangement of metal nanoparticles or nanostructures within nanometer scale inter-particle distance [13].

Electrodeposition of metal nanostructures is one of the simple, cost effective and efficient approaches that realize the defect-free packing of nanostructures over a wide area [14]. To enhance the selectivity within SERS, recognition molecules that specifically bind to targets can be immobilized on the SERS substrate. However, the size and length of the recognition molecule should not be very large otherwise the SERS effect may completely diminish due to the long distance between the captured protein and the plasmonic surface. In other words, the binding receptor should not be far away from the surface as SERS signal exponentially decreases with respect to the increase in distance between the surface and analyte.

Antibodies are frequently used as recognition molecules for detecting proteins. However, antibodies usually have large size that constitutes a serious hurdle to the label-free SERS detection of proteins [7]. Aptamers are now widely emerging as better choice over antibodies [15]. Aptamers are of much smaller size than antibodies and also well-known for their high selectivity, binding affinity, easy and quick production, stability and cost-effectiveness [16]. Moreover, aptamers can bend and orient themselves close to the surface of the SERS substrate after binding with the target protein [17]. This orientation would lead to high intensity SERS signal due to short distance between the captured target and SERS surface. Thus, this article presents a homogeneous and sensitive aptamer-functionalized nanosensor for the rapid reproducible and label-free SERS detection of rHuEPO in biological fluids.

## Methods

Nanostructured SERS substrate (pAu/AuNS) was prepared by potentiostatic deposition of AuNS over mirror polished Au surface [14] (see Additional file 1).

## Results and discussion

### Characterization of SERS substrate

**Figure 1:**
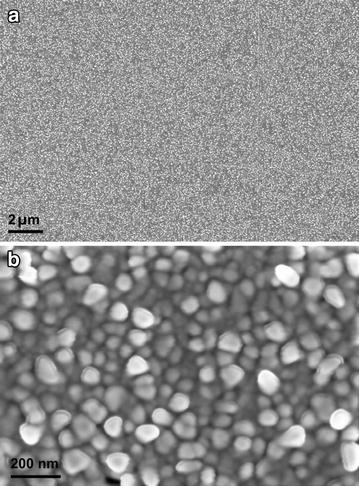
**a**–**b** SEM images of pAu/AuNS surface under different magnifications.

We optimized gold chloride concentration, electrolyte, applied potential and time to have a closely packed single layer of AuNS within nanometer scale inter-particle distance. Figure 1 shows the SEM pictures of pAu/AuNS surface. The SEM image under wider magnification (Figure 1a) illustrates that the deposition of AuNS is virtually uniform over the entire surface. Even at a 100 micron field of view, the particle coverage was uniform and defect free (Additional file 1: Figure S1). Similar SEM images were obtained over the entire 8 mm diameter pAu/AuNS disc surface which clearly reveals that the electrodeposition method produced homogeneous AuNS over the entire surface. Although the sizes of AuNS ranged between 10 and 100 nm (Figure 1b), the uniform close packing of AuNS in single layer lead to homogeneous SERS signal for a focused micron-scale laser spot [11] (vide infra). Polished Au was chosen as the underlying support since it produces more AuNS particle initiation spots during electrodeposition in contrast to glassy carbon or indium tin oxide surfaces [14]. As a result, high density of small-sized and closely packed AuNS was produced and led to enormous SERS enhancement. Furthermore, a possible coupling between the propagating surface plasmon polariton (SPP) of the underlying polished Au surface and surface plasmon resonance (SPR) of the AuNS [14, 18] may lead to additional SERS enhancement.

### Reproducibility of SERS spectra

**Figure 2:**
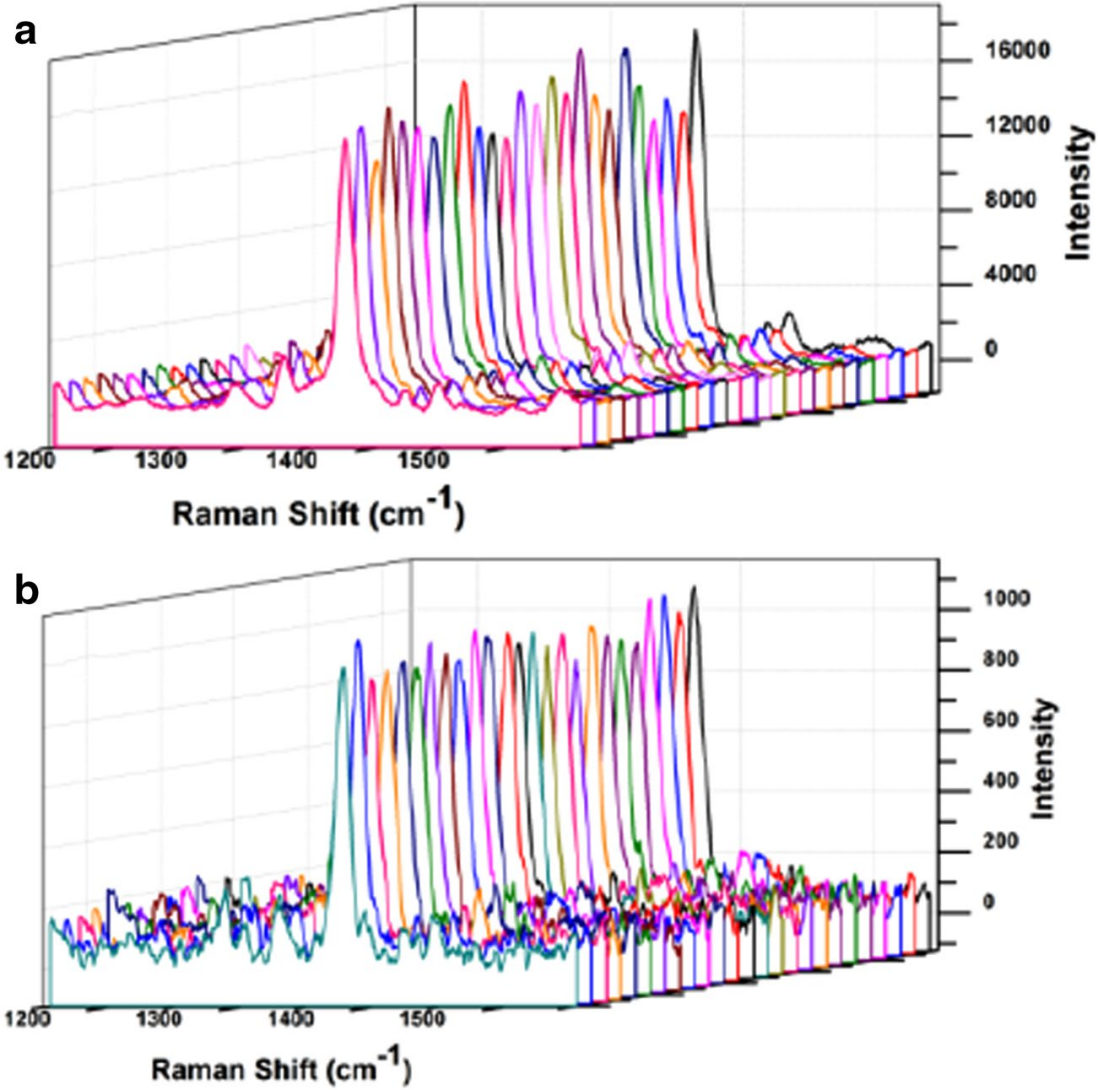
A series of SERS spectra of 2-QT randomly collected over the entire 8 mm diameter pAu/AuNS disc using **a** 50× objective with a laser focusing area of 0.32 µ^2^ and **b** 5× objective with a laser focusing area of 12.56 µ^2^.

In order to test the homogeneity of the substrate and the reproducibility of the SERS signal from various locations on the sensor, we used 2-quinolinethiol (2-QT) as a probe molecule due to its large Raman scattering cross section. Additional file 1: Figure S2 depicts the SERS spectrum of the self-assembled monolayer of 2-QT over pAu/AuNS substrate. The intense band at 1,371 cm^−1^, corresponding to the aromatic ring stretching, was used to calculate the relative standard deviation (RSD) of the SERS signal intensity from various surface locations. For comparison purposes, we carried out the SERS measurements using used 5× (spot diameter = 3.99 µm; illuminated area = 12.56 µ^2^ and working distance = 14 mm) and 50× (spot diameter = 0.64 µm; illuminated area = 0.32 µ^2^ and working distance = 0.37 mm) objectives respectively. When the laser beam is focused using the 50× objective, the RSD of the SERS signal at 1,371 cm^−1^ (150 measurements from various surface spots, Figure 2a) was found to be 8.74%. However, the RSD reduces to 4.92% when using the 5× objective (Figure 2b). This decrease in RSD with increase in laser focusing area (5× objective has 39.25 times higher illumination area than 50× objective) is rational as the acquired SERS signal is highly averaged when increasing the focusing area.

Using a 5× objective to focus the laser beam onto the pAu/AuNS substrate creates a wide area illumination (WAI) setting which allows for an increased area of the pAu/AuNS surface to be probed when compared to the area probed by the 50× objective. The increase in the area probed by the laser excitation beam contributes to the reproducibility of the SERS signal [19, 20]. This is confirmed by the low RSD of 4.92% obtained when using the 5× objective. In other words, the WAI setting allows for averaging the SERS signal from a large surface area of the aptasensor and hence the low RSD of the SERS measurements. Also the homogeneity of the particle coverage and packing leads to homogeneous distribution of the hot spots to give reproducible SERS signals despite the polydispersity (10–100 nm) of AuNS.

### Selective extraction and SERS investigation of rHuEPO in aqueous medium

**Figure 3:**
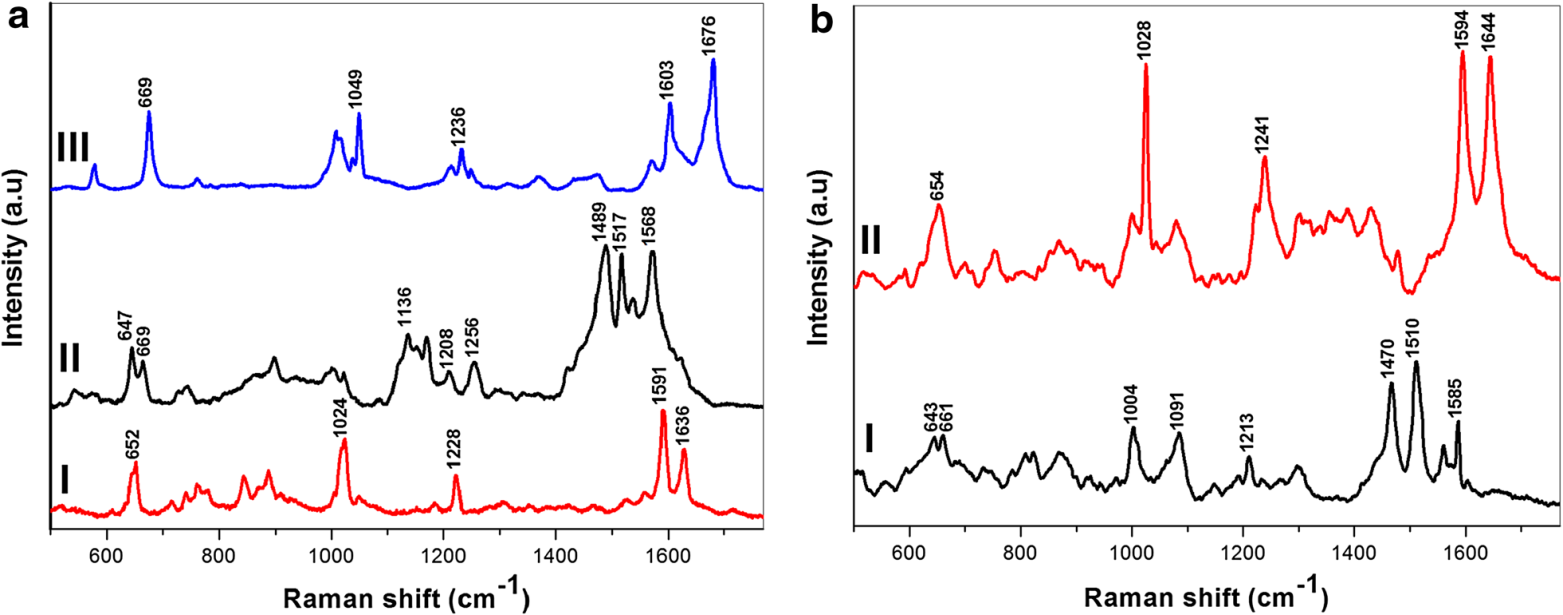
SERS spectra of **a** *I* pAu/AuNS/rHuEPO (drop dry), *II* pAu/AuNS/Apt (self-assembled monolayer) and *III* pAu/AuNS/Apt/rHuEPO (selectively captured). **b** SERS spectra of pAu/AuNS/Apt incubated in *I* blank horse plasma and *II* rHuEPO spiked horse plasma. Each spectrum is the average of 10 spectra.

The developed pAu/AuNS substrate was then functionalized with EPO-specific aptamer and the remaining bare sites on the surface backfilled with 6-mercapto hexanol (see Additional file 1) to create the aptasensor. The aptamers-functionalized nanosensor was then used for the selective capturing of rHuEPO from aqueous medium followed by the direct SERS detection of the captured protein. To acquire the native Raman fingerprint of rHu-EPO, we dropped 10 nM rHuEPO over a pristine pAu/AuNS surface and allowed it to dry in inert atmosphere. The subsequent SERS spectrum rHuEPO over non-functionalized surface is depicted in Figure 3aI. It is well known that the amide and aromatic (phenylalanine, tyrosine, tryptophan and histidine) vibrational bands dominates the Raman spectrum of proteins and polypeptides [21–27]. Similarly, the SERS spectrum of pAu/AuNS/rHuEPO (Figure 3aI) showed intense bands at 1,636 and 1,228 cm^−1^ corresponding to amide I and amide III vibrational modes, respectively [21–27]. The amide I at 1,636 cm^−1^ represent unique fingerprint of proteins as does not overlap with other vibrational modes from other functional groups. Therefore it can be used as a marker band for the identification of proteins without any external Raman labelling [28, 29].

**Scheme 1:**
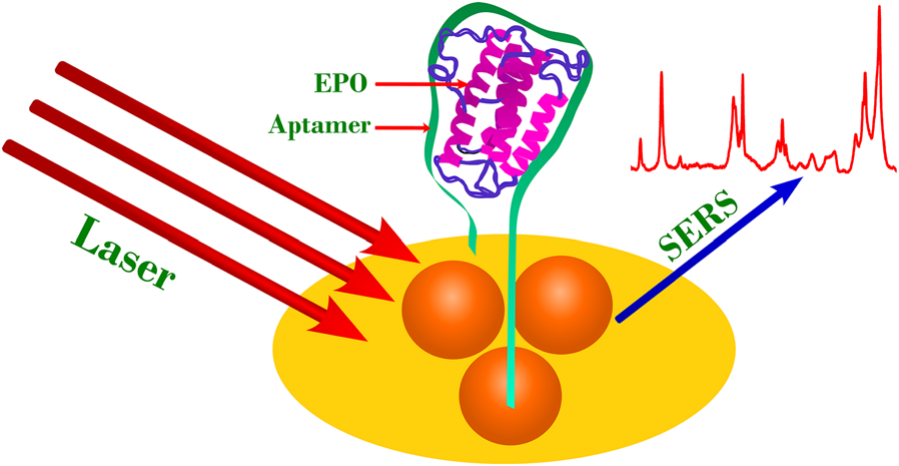
Graphical representation of rHuEPO captured aptasensor and subsequent SERS investigation.

The bands at 1,591 and 1,024 cm^−1^ correspond to the aromatic amino acid vibrational modes. Figure 3aII depicts the SERS spectrum of EPO aptamer on nanostructured surface. The SERS spectrum of an aptamer is usually dominated by adenine and guanine as the order of SERS cross–section is adenine > guanine > cytosine > thymine [30, 31]. Figure 3aII clearly indicated that the SERS spectrum of aptasensor is heavily dominated by the guanine vibrational modes at 647, 1,489 and 1,568 cm^−1^. This is because the EPO aptamer has higher contribution from guanine nucleobase than adenine (13:5). Also, the C-S stretching mode for the thiolated aptamer is depicted at 669 cm^−1^. Figure 3aIII shows the SERS spectrum of the apatsensor after capturing the rHuEPO protein (pAu/AuNS/Apt/rHuEPO) on its surface by the EPO aptamers. Scheme 1 depicts the graphical representation of rHuEPO captured aptasensor and subsequent SERS investigation. SERS The strong band at 1,676 cm^−1^ is attributed to the amide I vibrational mode [21–27]. The amide I band at 1,676 cm^−1^ is red shifted by 40 cm^−1^ when compared to that of native rHuEPO at 1,636 cm^−1^ (Figure 3aI). Similarly, the vibrational modes of the aromatic amino acids at 1,603, 1,236 and 1,049 cm^−1^ are also red shifted. The shift in vibrational energy of the rHuEPO over the aptasensor surface is attributed to the aptamer conformational rearrangements upon binding rHuEPO to the aptamer fragment antigen binding (Fab) regions in the aqueous medium [7, 32].

**Figure 4:**
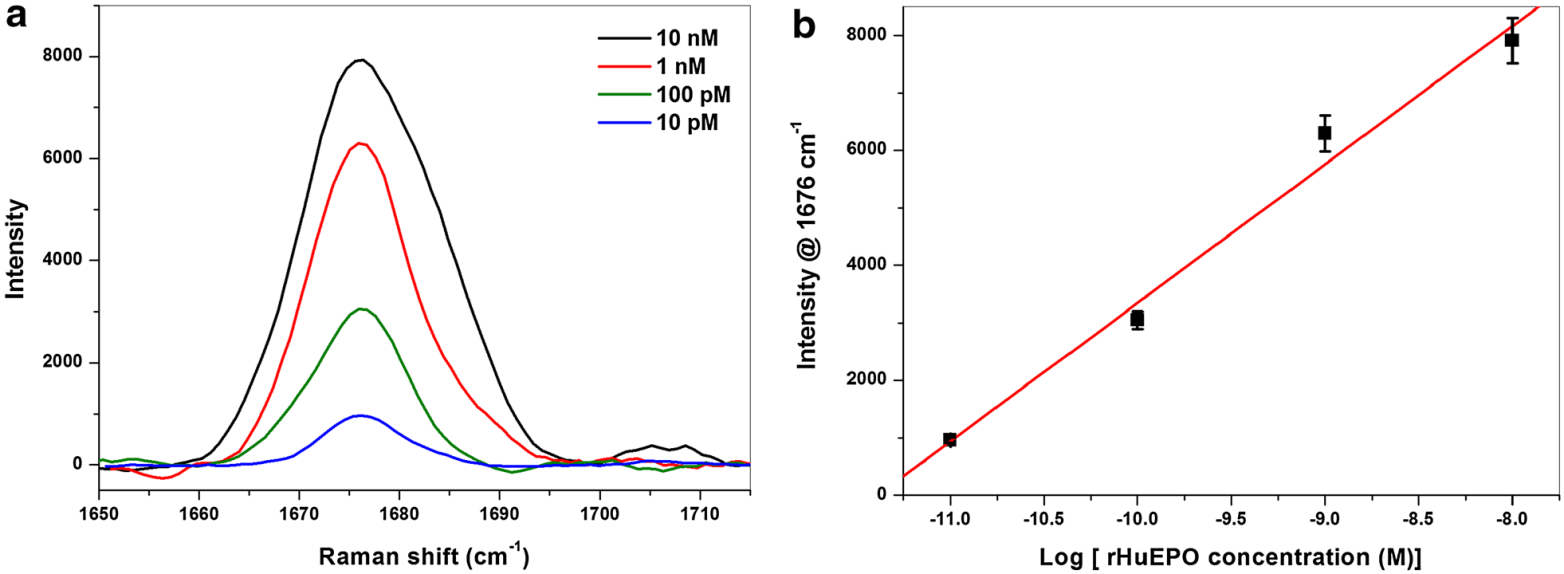
**a** SERS spectra of pAu/AuNS/Apt/rHuEPO with decreasing concentration of rHuEPO: 10 nM (*black*), 1 nM (*red*), 100 pM (*green*), and 10 pM (*blue*). **b** Plot demonstrating the linear relationship between log concentration of rHuEPO and SERS intensity at 1,676 cm^−1^.

In order to reveal the suitability of the present aptamer modified pAu/AuNS surface towards SERS quantification of rHuEPO, various concentrations of rHuEPO in aqueous medium were employed. To each concentration of rHuEPO, a freshly prepared pAu/AuNS/Apt surface was used to selectively capture rHuEPO onto the aptasensor surface and subsequently screened under the Raman microscope. Figure 4a shows the SERS spectra (amide 1 vibrational mode) of the pAu/AuNS/Apt/rHuEPO surface within a rHuEPO concentration range of 10 nM to 10 pM (the spectra were normalized and background subtracted). The band centered at 1,676 cm^−1^ corresponding to amide I vibrational mode of rHuEPO was used a reference band for rHuEPO quantification. The SERS signals were found to monotonically decrease with decreasing concentration (Figure 4a). A linear relationship was obtained between the SERS signal intensity at 1,676 cm^−1^ and the corresponding rHuEPO log concentration plot as depicted in Figure 4b. Similar linear relationship between log(concentration) and SERS intensity was formerly demonstrated in the literature [8, 33]. As indicated by Figure 4b, good correlation (*R*^2^ = 0.993) was found over a wide concentration range of TNT (10^−8^ to 10^−11^ M).

### Selective extraction and detection of rHuEPO in biological fluids

As a proof of concept for the SERS detection of EPO doping in biological fluids, we extended our methodology towards the label-free identification of rHuEPO in horse plasma. 1 nM rHuEPO was spiked into neat horse plasma and subsequently dropped over the pAu/AuNS/Apt. After 30 min, the substrate was washed with Millipore water to remove the biological matrix and dried in gentle flow of argon gas. A similar blank experiment was also carried out using un-spiked serum. Figure 3bI, II show the SERS spectra of blank and rHuEPO spiked (Figure 3bII), on aptasensor respectively. Figure 3bI shows strong correlation to Figure 3aII of the pAu/AuNS/Apt before interaction with EPO protein. The resemblance between Figure 3aII, bI indicates that the aptasensor when interacted with blank plasma matrix (no EPO spiked in matrix) did not bind with any of the non EPO proteins that exist in horse plasma. This result confirms the selectivity of the aptasensor towards its target protein over other proteins that may co-exist in a biological matrix. This Figure 3bII clearly depicted the amide I band at 1,644 cm^−1^ and aromatic amino acid vibrations at 1,594 and 1,028 cm^−1^. A close comparison between Figure 3aI, bII confirms unambiguous resemblance between the spectrum of rHuEPO standard and that of the aptasensor after interacting with the plasma sample spiked with rHuEPO. The shifts in band positions between Figure 3aIII, bII may attributed in part to the higher dielectric constant and solvent polarity of aqueous matrix in comparison to those of the protein rich plasma matrix [34]. In Figure 3aIII, the higher dielectric constant and polarity of the aqueous matrix may have caused the red shift of the amid I band to the higher wave number of 1,676 cm^−1^ from its position at 1,636 cm^−1^ in Figure 3aI, bII. Therefore, we have successfully demonstrated the selective extraction and SERS identification of rHuEPO in horse plasma. This outcome clearly indicates the significance of using aptamers-functionalized homogeneous SERS substrate for facile and rapid screening of proteins in biological fluids. The full potential of the aptasensor is realized when it is combined with handheld Raman device for the in-field detection of rHuEPO from biological matrices.

## Conclusion

We demonstrated a sensitive and homogenous aptasensor for the reproducible detection of EPO in biological fluids. Due to the close packing and homogenous distribution of the nanoparticles coverage over the surface, strong and reproducible signal enhancement is acquired especially under WAI conditions where the RSD of the SERS measurements can be as low as 4.92%. By adapting to wide area, handheld Raman devices can used in combination with the new aptasensor for the label-free in-field screening of rHuEPO in horse plasma.

## Additional file

**Additional file 1.** Experimental producers to fabricate reproducible aptamer-functionalized SERS substrate as well as the detection of EPO in horse plasma are given in this document.

## Abbreviations

EPO: erythropoietin
rHuEPO: recombinant human EPO
SERS: surface enhanced Raman spectroscopy
AuNS: gold nanostructures
pAu/AuNS: gold nanostructures over a gold substrate
aptasensor: aptamer-functionalized biosensor
WAI: wide area illumination
2-QT: 2-quinolinethiol
