# Supplementary material for: Reproducible and label-free biosensor for the selective extraction and rapid detection of proteins in biological fluids
Source: J Nanobiotechnology. 2015 Jun 24;13:43. doi: 10.1186/s12951-015-0102-8 (PMC4477471; doi:10.1186/s12951-015-0102-8)
Supplement: Supplementary file 1 — Additional file 1. Experimental producers to fabricate reproducible aptamer-functionalized SERS substrate as well as the detection of EPO in horse plasma are given in this document. [file 12951_2015_102_MOESM1_ESM.doc]

**Reproducible and label-free biosensor for the selective extraction and rapid detection of proteins in biological fluids**

Arumugam Sivanesan^a^*, Emad L. Izake^a^*, Roland Agoston^a^, Godwin A. Ayoko^a^, Martin Sillence^b^

^a^ Nanotechnology and Molecular Sciences Discipline, Faculty of Science and Engineering, Queensland University of Technology, 2 George St., Brisbane, QLD 4001, Australia

^b^ Discipline of Biosciences, Faculty of Science and Engineering, Queensland University of Technology, 2 George St., Brisbane 4001, QLD, Australia

E-mail address: [sivanesan.arumugam@qut.edu.au](mailto:sivanesan.arumugam@qut.edu.au); [asnesan@gmail.com](mailto:asnesan@gmail.com) (A. Sivanesan)
 [e.kiriakous@qut.edu.au](mailto:e.kiriakous@qut.edu.au) (E. L. Izake)

Experimental producers to fabricate reproducible aptamer-functionalized SERS substrate as well as the detection of EPO in horse plasma are given in this document.

**Experimental Details**

**1. Chemicals and Materials**

Hydrogen tetrachloroaurate (HAuCl_4_.4H_2_O) and 6-mercaptohexanol were purchased from Sigma Aldrich (USA). The recombinant human erythropoietin (rHuEPO) specific aptamer sequence (5′-HO-S-S-(CH2)6-TTGAAAGGTCTGTTTTTGGGGTTGGTTTGGGTCAA-3') was synthesized by FRIZ Biochem (Neuried, Germany). Tris(2-carboxyethyl)phosphine (TCEP) was purchased from Pierce. All other chemicals were of Analytical grade. All dilutions were made using deionised water (18.2 MΩ.cm) from a Millipore water purification system. Polycrystalline gold discs (Au) having a geometric area of 0.502 cm^2^ and platinum wire (A & E Metals, Australia) were respectively, used as working and counter electrode. Dry leakless electrode (DRIREF-2, World Precision Instruments, USA) was used as a reference electrode. Polishing slurries and pads (Microcloth®) were purchased from Buehler, Germany.

**2. Instrumentation**

All electrochemical experiments were carried out in Autolab PGSTAT204 potentiostat with a custom-made three-electrode cell setup. All Raman measurements were performed using the Renishaw InVia Raman microscope equipped with 785 nm laser line as excitation source. Spectra were collected using a 50× and 5× objective lens over a wavelength range from 500 cm^-1^ to 2000 cm^-1^ using a laser power of 1 mW for 10s (3 accumulations). For each Aptamer and rHuEPO spectrum, 10 spectra were randomly recorded over the entire surface and averaged. SEM measurements were performed using Zeiss Sigma VP Field Emission Scanning Electron Microscope with an accelerating voltage of 5 kV under high vacuum.

**3. SERS Substrate Preparation**

Au disc electrodes were manually mirror-polished with alumina slurries of sequentially decreasing particle sizes (0.5 µm, 0.05 µm and 0.02 µm). After each step of polishing, the electrodes were immersed in Millipore water and subsequently sonicated in an aqueous ultrasonic bath for 15 minutes in order to remove the physically adsorbed alumina particles from the electrode surface. Prior to the deposition of gold nanostructures, the Au discs were cleaned by immersing into piranha solution for 10 min (3:1, 98% H_2_SO_4_ / 30% H_2_O_2_) and subsequent thorough washing with copious amount of Millipore water. *Warning*: piranha solution is very corrosive and must be handled with extreme caution; it reacts violently with organic materials and should not be stored in tightly closed vessels.

Nanostructured SERS substrate was prepared by potentiostatic deposition of AuNS over flat Au surface (pAu/AuNS). The three-electrode electrochemical cell was filled with the solution of 4 mM HAuCl_4_ in 0.1 M HClO_4_ and subsequently purged with high pure argon gas for 30 min to remove oxygen from solution. A potential of -80 mV was applied for 400 s and then the electrode was removed from the solution and subsequently washed with Millipore water to remove other ions from the surface. The electrode was then dried under a stream of nitrogen gas and used as a SERS substrate.

**4. Fabrication of aptasensor and rHuEPO capture**

The aptamers were received in lyophilized form and dissolved in selection buffer (20 mM Tris-HCl, 140 mM NaCl, 5 mM MgCl2 and 5 mM KCl at pH 7.5) to have a stock concentration of 100 µM. Prior to fabricate the aptamer over pAu/AuNS surface, 100 µL of 0.2 µM aptamer was pre-treated with 100 fold excess of tris(2-carboxyethyl)phosphine (TCEP) for 1 h at room temperature to reduce the disulfide bond of the aptamer. The pAu/AuNS substrate was then incubated in Aptamer-TCEP mixture for overnight at room temperature. Followed by the substrate was washed with selection buffer and copious amount of Millipore water to remove the unbounded aptamer from the surface. The surface was then dried in a gentle stream of argon and immediately transferred into 2 mM 6-mercaptohexanol (6-MH) in selection buffer and allowed to stand for 3 h. Subsequently, the substrate was washed with selection buffer and Millipore water to remove all the unbounded 6-MH from the pAu/AuNS surface. Now the aptasensor (pAu/AuNS/Apt) is ready to capture rHuEPO. The sensor is then incubated into 1 nM rHuEPO in Tris buffer (pH 7.5) for 1 h and subsequently washed in Millipore water to remove the free rHuEPO from the sensor surface. The aptasensor binding to rHuEPO ((pAu/AuNS/Apt/rHuEPO) was then dried in gentle flow of argon prior to SERS measurements.

**5. Horse Serum Experiment**

Blank horse plasma samples were donated by Dr. Rohan Steel, Project Leader, Biological Research Unit, Racing Analytical Services Ltd, Melbourne. The samples were collected under the Melbourne lab protocols and ethical clearances, arrangements and protocols are all maintained by this lab. The samples were also shipped under their lab protocols for shipping biological specimens. The samples were received at QUT and used as matrix for Raman testing. This did not require PC2 lab at QUT.

rHuEPO was spiked into the horse serum to obtain a final concentration of 1 nM rHuEPO. The aptasensor was then incubated in the rHuEPO spiked horse serum for 1 h and subsequently washed to copious amount of water to remove the biological matrix and unbounded rHuEPO from the sensor surface. Finally, the substrate was dried in gently flow of argon and used for SERS measurements.


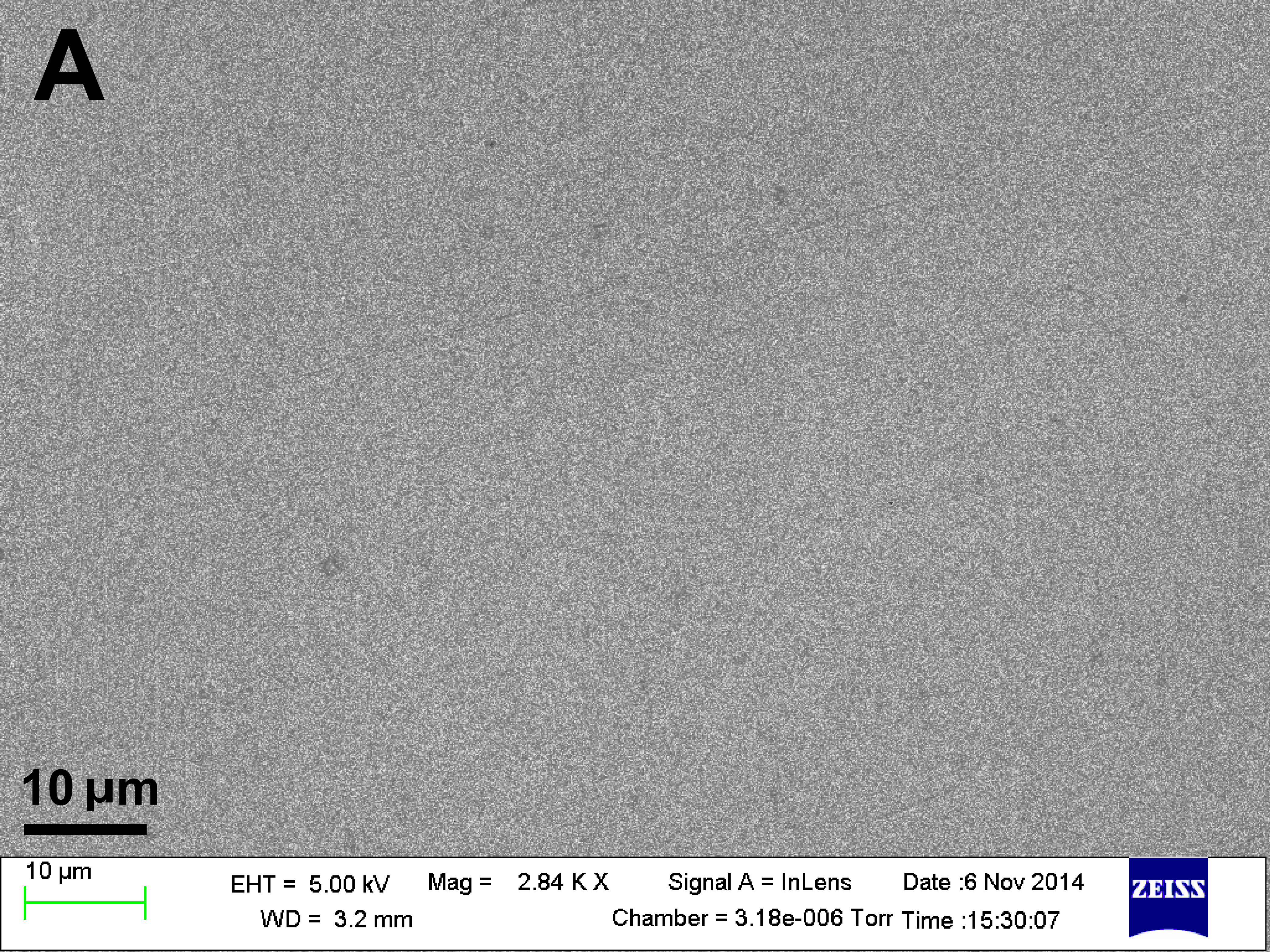

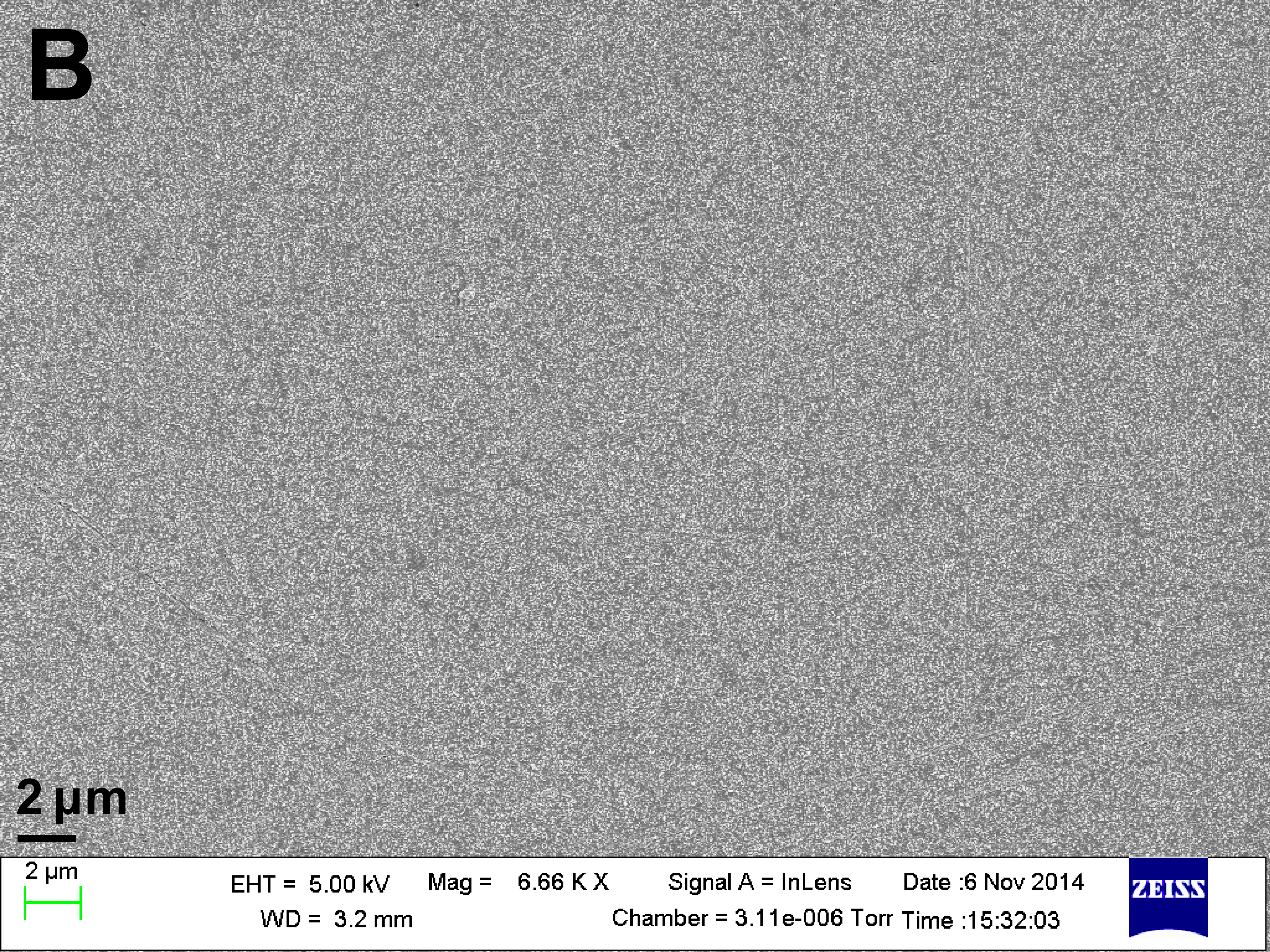


**Figure S1**. SEM images of pAu/AuNS surface under wider magnification

**
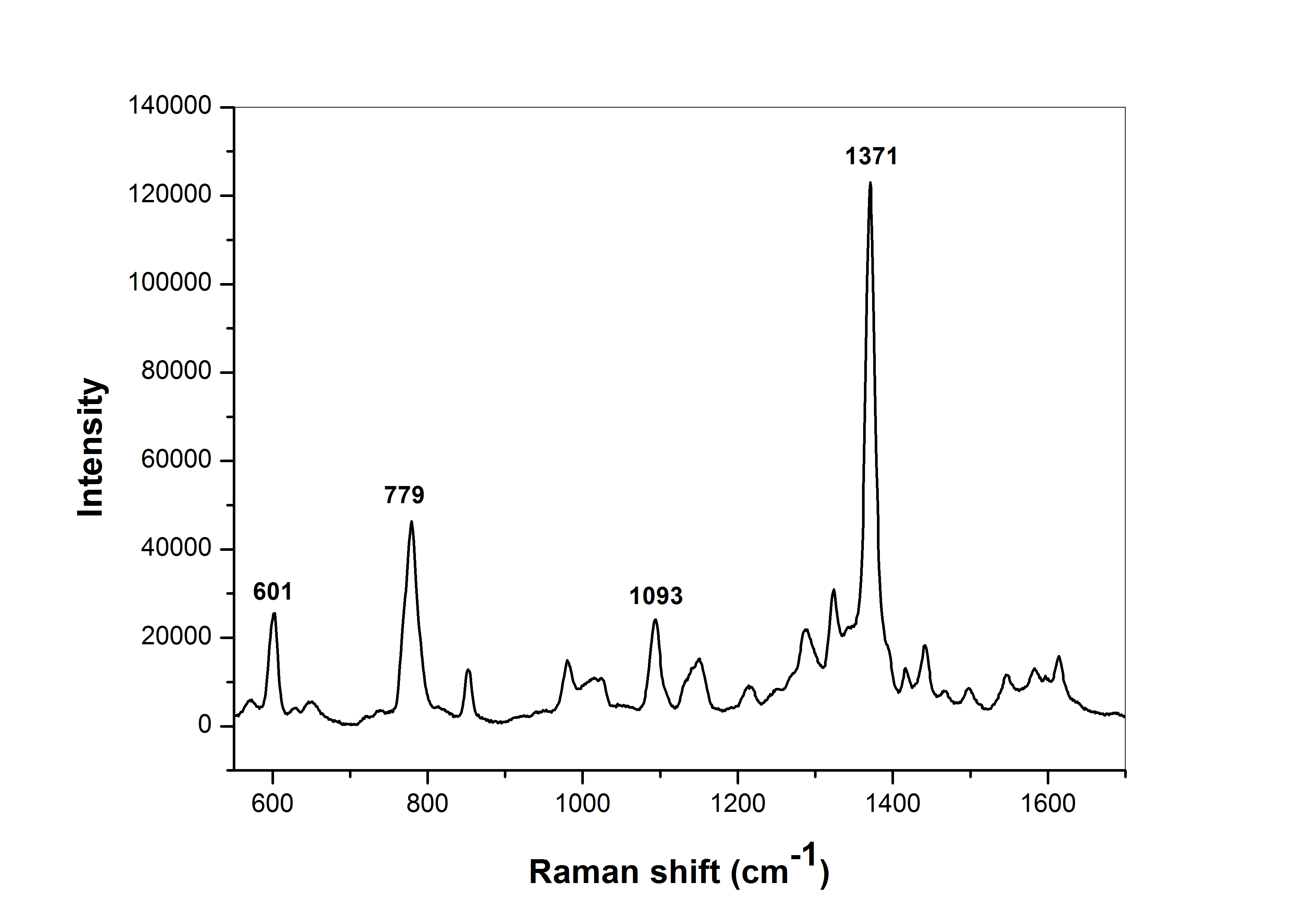
**

**Figure S2**. SERS spectrum 2-QT monolayer on pAu/AuNS surface.

1.Z. Zhang, L. Guo, J. Tang, X. Guo and J. Xie, Talanta, 2009, 80, 985-990.

2. J. Sun, A. Guo, Z. Zhang, L. Guo and J. Xie, Sensors, 2011, 11, 10490-10501.
